# National dissemination of an online research mentor training intervention: Evidence of an asynchronous model to promote learning outcomes and behavior change

**DOI:** 10.1017/cts.2025.84

**Published:** 2025-04-28

**Authors:** Anne Marie Weber-Main, Kimberly Spencer, Emma Dums, So Hee Hyun, Christine Pfund

**Affiliations:** 1 Department of Medicine, University of Minnesota, Minneapolis, MN, USA; 2 Clinical and Translational Science Institute, University of Minnesota, Minneapolis, MN, USA; 3 Institute for Clinical and Translational Research, University of Wisconsin–Madison, Madison, WI, USA; 4 Center for the Improvement of Mentored Experiences in Research, University of Wisconsin– Madison, Madison, WI, USA

**Keywords:** mentoring, professional development, asynchronous, mentor training, workforce development

## Abstract

Engaging, accessible, evidence-based interventions are needed to support the professional development of research mentors within the clinical and translational sciences. This article reports on the usage and impact of the University of Minnesota Clinical and Translational Science Institute’s online mentor training module, *Optimizing the Practice of Mentoring (OPM).* Among the 1,124 *OPM* users in our contemporary evaluation sample (Feb 2019–June 2022), retrospective pre-to-post gains were observed in respondents’ self-rated mentorship skills (11 items), perceptions of the overall quality of mentoring they provide, and mentoring confidence. A high proportion (83%) of users reported making or intending to make changes in their mentoring practices as a result of the training. Example behavior changes included a greater focus on aligning expectations, more proactive attention to the relationship (overall and its distinct phases), increased usage of active communication skills, adoption of tools such as Individual Development Plans, and ongoing self-reflection. Over a 10-year period, 4,011 unique individuals registered for the module, representing over 650 different institutions (a majority being affiliated with past or current Clinical and Translational Science Hubs). *OPM* has the versatility to be employed as a standalone, asynchronous approach for mentor development or as one curricular component of more comprehensive, multimodal programs.

## Introduction

Effective mentorship plays a critical role in the long-term persistence and academic success of trainees in research career pathways, having a significant impact on mentees’ research productivity, academic and research self-efficacy, and career satisfaction [1–7]. However, trainees from historically and systemically excluded groups are less likely than others to be in effective mentoring relationships [2,4,7–12]. This knowledge has contributed to a burgeoning national focus on mentorship over the past decade and resulted in funding agencies requiring mentorship plans – and in some cases mentorship education (mentor and mentee training) – to improve the effectiveness of these relationships [13–15].

This article reports on the impact and national usage of one innovative approach to training research mentors: the asynchronous, self-paced, online module, *Optimizing the Practice of Mentoring (OPM)*. *OPM* was developed over a decade ago at the University of Minnesota and is currently maintained with support from its Clinical and Translational Science Institute (CTSI) [16]. The first version of the module was targeted to mentors of graduate students, postdoctoral fellows, and junior faculty who are engaged in biomedical research. Subsequently, a version adapted for mentors of undergraduate students was created.

**Figure 1.**
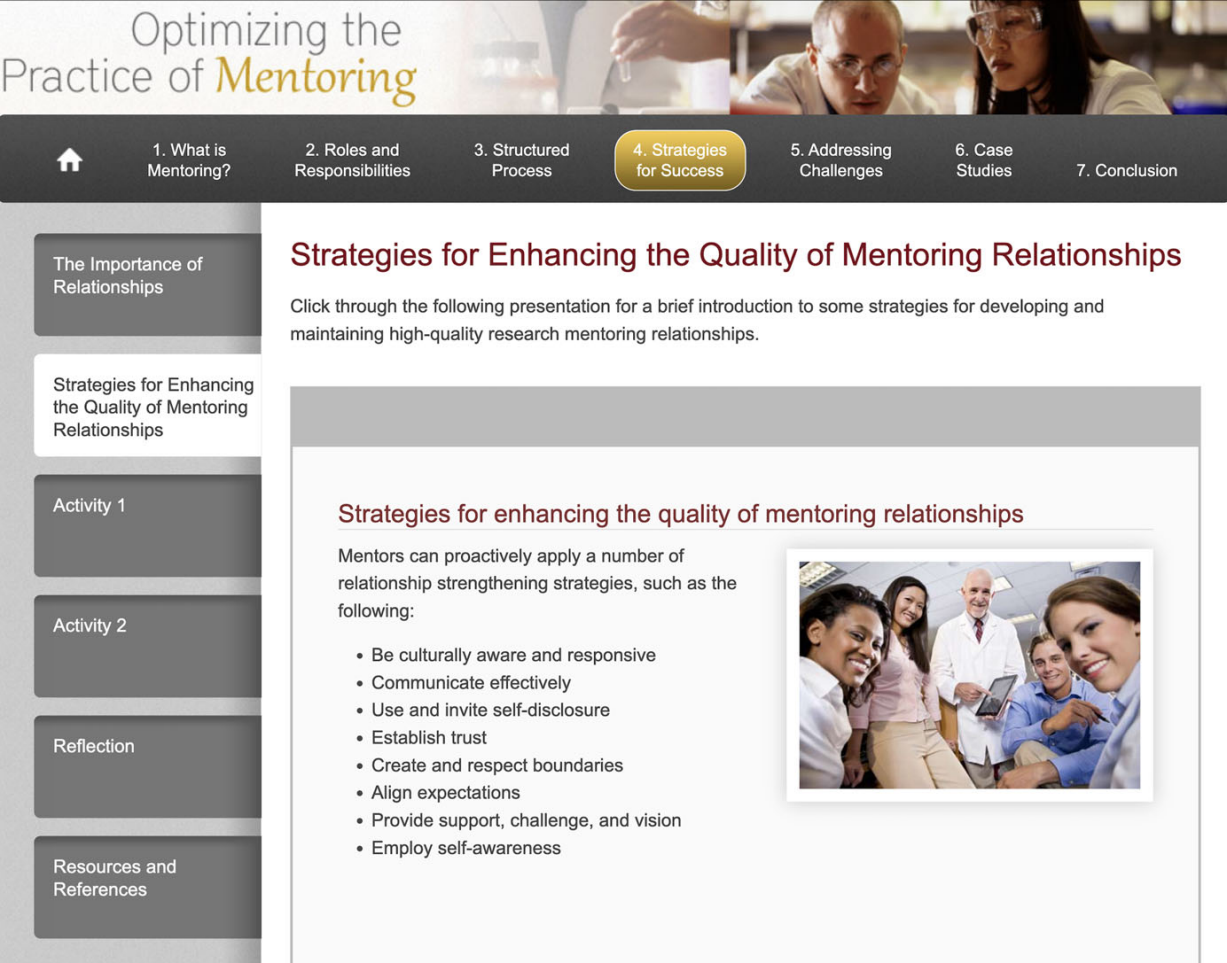
Screenshot from the *Optimizing the Practice of Mentoring* online training module. Module content is divided into the seven major sections shown in the top horizontal bar. Users navigate to specific content within a section by clicking on the labeled tabs on the left side of the screen.

*OPM* content provides users with a foundational introduction to research mentorship. The module begins by describing the empirically demonstrated value of mentorship and the diverse ways it can be implemented (such as dyadic, group, and peer mentoring models). Section 2 of the module provides an example-laden overview of research mentors’ roles and responsibilities within the career and psychosocial domains of mentorship. In Section 3, users are provided with tips and tools to proactively attend to the four developmental phases of a mentoring relationship (preparation, negotiation, cultivation, and closure). Section 4 introduces some key strategies for developing and maintaining successful mentoring relationships, such as establishing trust, aligning expectations, offering mentees a combination of support and growth-focused challenges, and engaging in routine self-reflection. These strategies are reinforced in Sections 5 and 6 through case studies that highlight specific mentorship challenges. Users are prompted to reflect on these challenges and consider approaches for preventing and addressing them. *OPM* engages users through text, audio, mini-presentations, case studies, and brief interactive activities. Users also have access to a tool kit of resources and the option to create and email to themselves a mentoring action plan. A screenshot illustrating the overall organization of *OPM* content is provided in Figure 1.

In 2019, *OPM* was updated with financial support from the National Institutes of Health’s National Research Mentoring Network (NRMN). This work was completed in partnership with investigators at the University of Wisconsin–Madison and other national collaborators affiliated with NRMN’s Mentor Training Core. Much of the module’s content was refreshed, with greater attention given to building cultural awareness in mentoring. Other improvements included changes in the registration platform, more streamlined curation of resources, addition of self-reflection questions, inclusion of a research self-efficacy exercise curated from a published module [17], and a new embedded evaluation survey. The version of *OPM* adapted for mentors of undergraduate students was also created at this time.

Since its initial launch in 2012, *OPM* has been publicly available at no cost to users, requiring only the creation of a University of Minnesota guest email account for registrants from other institutions. However, the full extent of the module’s reach has not been documented, and published data on its impact are limited. In 2019, findings were published from a pilot randomized controlled trial of the University of Minnesota CTSI’s Mentoring Excellence Training Academy [18]. This professional development program consists of two components: completion of the *OPM* online module, followed by 5 hours of in-person facilitated workshops based on the well-studied *Entering Mentoring* curriculum [19–23]. The workshop topics covered in the Academy included the following: maintaining effective communication, aligning expectations, addressing equity and inclusion, fostering independence, and promoting professional development. The Academy’s hybrid training model reflects how we envisioned *OPM* would most commonly be used – as a didactic, asynchronous, introductory module that provides mentors with foundational information about mentorship (its working definition, implementation models, core functions, and stages of relationship development) and introduces some of the many research mentorship competencies that might be more deeply explored in a subsequent workshop setting. The pilot trial [18] of the Academy demonstrated significant mentorship skills gains at 3-month follow-up for participants who completed the full hybrid training program. But the trial also generated promising preliminary evidence that *OPM* has significant value when used independently – that is, as a standalone training module. Specifically, mentors who engaged with *OPM’s* online, interactive material reported greater knowledge gains than mentors in the control arm who received only a simple written summary of the module’s content. Additionally, for 42% of mentors in the trial’s intervention arm, *OPM* completion alone (before any engagement in the synchronous workshop component) was sufficient to prompt an intention to make changes to their current mentoring practices.

This article builds on these early preliminary results of *OPM’s* impact. Our objective was to more comprehensively evaluate outcomes for *OPM* when implemented as a solo, asynchronous, self-paced intervention for mentor development. For the current analysis, we examined over 3 years of evaluation data collected from a large national sample of *OPM* users (specifically, the updated 2019 version for mentors of graduate students, fellows, and faculty). We used a retrospective pre-post survey design to assess users’ perceived changes in skills that reflect the module’s learning objectives, as well as changes in their self-rated quality of mentoring provided, confidence in mentoring, and confidence in meeting mentees’ expectations. We report on users’ satisfaction with the module and their intent to change behavior as a result of completing the *OPM* training. Additionally, we document the scope of *OPM’s* national dissemination during its first full decade of availability and cite examples of how *OPM* can be integrated as a component of a more comprehensive mentor development program.

## Materials and methods

### Assessment of module outcomes

#### Participant sample

To assess *OPM’s* impact on learner outcomes, we analyzed data for individuals who registered for the module from February 19, 2019 (when the updated module and its new evaluation survey were first made available) through June 1, 2022 (the selected cutoff date for this analysis). During this time period, there were 2,023 unique registrants. Of these, 1,298 (64%) submitted the module’s evaluation survey. We excluded 174 individuals who indicated they were a postdoctoral fellow, graduate student, or undergraduate student, because the module was designed for mentors of these types of trainees. Although some fellows and graduate students do serve as ancillary mentors (typically for undergraduate students), we reasoned that their exclusion from this analysis would better enable us to assess the module’s effectiveness among its intended target audience. Therefore, our final analysis sample consisted of 1,124 people.

#### Data collection

The data analyzed for this report were collected under a University of Wisconsin–Madison IRB exempt protocol (#2017-0026). Following completion of *OPM*, users are asked to complete an evaluation survey in Qualtrics (online survey platform). The survey collects information on *OPM* users’ demographic characteristics and professional backgrounds, including their previous mentoring experience and training. Respondents are asked to indicate how much time they spent engaging with *OPM* (choice of half hour increments, ranging from “less than 1 hour” to “3.5 or more hours”). User satisfaction is measured by Likert-scale items asking whether respondents felt that participating in *OPM* was a valuable use of their time (*5-point scale, 1-Strongly disagree, 2-Disagree, 3-Neither agree nor disagree, 4-Agree, and 5-Strongly agree*) and how likely they were to recommend the module to others (*5-point scale, 1-Very unlikely, 2-Unlikely, 3-Undecided, 4-Likely, and 5-Very likely*).

Eleven survey items were developed to assess *OPM* users’ self-reported skill gains in content areas covered by the module (for example, “Recognizing the pros and cons of different mentoring models,” “Fulfilling the psychosocial functions of being a research mentor,” “Applying a proactive, structured approach to mentoring,” “Engaging in difficult conversations with my mentees”). For each item, users are asked to rate how skilled they feel they were before completing the training, and how skilled they feel they are now after completing the training (*7-point scale, 1-Not at all skilled, 4-Moderately skilled, 7-Extremely skilled*). The survey also includes three items that are routinely assessed across other mentor training programs, including those offered by the Center for the Improvement of Mentored Experiences in Research (CIMER; www.cimerproject.org). Those items are as follows: “Thinking back to before the training and now after the training: How would you rate the overall quality of your mentoring?” “How confident are you in your ability to mentor effectively?” (*7-point scale for each item, 1-Very low, 4-Average, 7-Very high*); “To what extent do you feel that you are meeting your mentees” expectations?” (*7-point scale, 1-Not at all, 4-Moderately, and 7-Completely).* Lastly, users are asked whether they made or are planning to make any changes in their mentoring relationships as a result of participating in the *OPM* training (*yes/no*). Those indicating *yes* are invited to describe their intended changes as an open-ended response.

#### Data analyses

We used paired t-tests to compare *OPM* users’ mean post-training scores to their mean retrospective pre-training scores for each outcome of interest: mentorship skills, quality of mentoring provided, confidence in mentoring, and confidence in meeting mentees’ expectations.

We applied an iterative coding methodology [24] to analyze free-text responses to the question asking respondents to describe changes they made or plan to make in their mentoring relationships. As a first step, an NVivo word frequency query function was used to identify the most frequently occurring words within the dataset. The query included words with the same stem (e.g., *plan, planned, planning*). Stem words included in the free-text response of at least 50 unique individuals were considered as possible coding categories. One word, *relationship*, was used by more than 50 individuals but was excluded from further analysis because it largely overlapped with another category. A few words that fell below this frequency threshold were also examined (*proactive, support*) because of their close alignment with the module’s content. The criterion of 50 unique respondents was used as a cut off, because it represented 1% of the weighted responses and because most terms used less frequently were overlapping with selected words or were not linked to specific behaviors (e.g., *clearly, ask, improve*). If an individual respondent mentioned two different stem words, that individual was counted in both stem counts.

As a second step, responses containing the identified stem word or similar words were reviewed by two authors (CP, KS) and used to develop a coding definition that reflected similarity in respondents’ meaning. We acknowledge that only including responses with the selected stems is a conservative approach to coding, but one that reduces the chance of misinterpreting responses. Using the final definitions, both authors independently read each response and employed focused coding to assign categories to each response. For example, for all responses that included the stem word *plan*, the authors reviewed entries for duplicates to ensure respondents were not counted more than once. The authors then reviewed entries to ensure they aligned with the coding definition and mentioned creating a written plan such as a mentoring agreement or individual development plan. The approach omitted entries that mentioned general planning (e.g., “I plan to communicate more” or “More formal plans and goals…”).

### Assessment of module dissemination

In addition to our analyses of *OPM* evaluation data for a 3-year period, we examined the full registration dataset extending back 10 years to characterize the national dissemination of *OPM* as a freely available, self-paced, online, asynchronous training intervention for research mentors. We assessed how many total people accessed the module during its first decade of availability (years 2012–2022), the extent of its reach both within and external to the University of Minnesota, and users’ motivations for participating in the training. Upon registering for *OPM*, users complete a brief online survey that collects their name, email, job title, institution, and reason for registering. Response options for the last item are as follows: “Required by my institution, department, or program;” “My own professional development;” “Reviewing module for possible use at my institution, department, or program;” and “Other.” Registration data are not linked to those acquired from the evaluation survey. The latter survey is anonymous and does not collect any identifying information.

## Results

### Module impact: evaluation data

#### Sample characteristics

Demographic and professional background characteristics of the *OPM* evaluation sample (*n* = 1,124) are summarized in Table 1. There was a nearly even split between those who self-identified as male and female, and 69% of those who reported on race selected White. Faculty participants at all academic ranks were represented. Lab-based research was the most common category for respondents’ research focus (61%), followed by translational research (20%) and clinical research (18%). *OPM* users exhibited diversity in their mentorship experience, both in terms of how long they had been a mentor (range of 0 to >20 years) and whom they were mentoring. The most common category of current mentees was graduate students (PhD or Master’s, 76%), followed by postdoctoral fellows (58%), undergraduate students (56%), and junior faculty (44%). The majority (58%) reported having had no prior research mentor training.

**Table 1.** Demographic characteristics and mentoring background of participants in *OPM* evaluation sample (February 19, 2019 through June 1, 2022)

Variable	Number	Percentage
**Gender** ^a^ **(*n* = 995)**		
Male	492	49.45%
Female	466	46.83%
Transgender	1	0.10%
Other	7	0.70%
Prefer not to report	34	3.42%
**Race** ^a^ **(*n* = 991)**		
American Indian or Alaskan Native	10	1.01%
Asian	184	18.57%
Black or African American	37	3.73%
Native Hawaiian or Pacific Islander	3	0.30%
White	688	69.42%
Other	30	3.03%
Prefer not to report	62	6.26%
**Hispanic or Latino** ^a^ **(*n* = 969)**		
Not Hispanic or Latino	849	87.62%
Cuban	3	0.31%
Mexican or Chicano	18	1.85%
Puerto Rican	12	1.24%
Other Hispanic or Latino	27	2.79%
Prefer not to report	63	6.50%
**Title** ^a^ **(*n* = 1080)**		
Dean	2	0.19%
Associate Dean	20	1.85%
Assistant Dean	9	0.83%
Professor	367	33.98%
Associate Professor	258	23.89%
Assistant Professor	401	37.13%
Scientist or Researcher	21	1.94%
Associate Scientist or Researcher	7	0.65%
Assistant Scientist or Researcher	4	0.37%
Clinical Instructor	19	1.76%
Lecturer or Instructor	8	0.74%
Training Program Director	19	1.76%
**Research focus** ^a^ **(*n* = 1077)**		
Behavioral research	101	9.38%
Clinical research	197	18.29%
Community engaged research	69	6.41%
Educational research	72	6.69%
Field/Applied research	80	7.43%
Lab-based research	661	61.37%
Social science research	69	6.41%
Theoretical research	64	5.94%
Translational research	216	20.06%
Other	42	3.90%
**Trainees currently mentoring** ^a^ **(*n* = 1077)**		
Senior faculty	73	6.78%
Junior faculty	478	44.38%
Postdoctoral fellows	625	58.03%
Clinical fellows	159	14.76%
PhD or Master’s students	818	75.95%
Medical or health care professional students	243	22.56%
Post Baccalaureate students	261	24.23%
Undergraduate students	602	55.90%
High school students	135	12.53%
K awardees	94	8.73%
T awardees	79	7.34%
Not currently mentoring trainees	29	2.69%
**Years of experience as research mentor (*n* = 1077)**		
0 years	50	4.64%
1 to 5 years	298	27.67%
6 to 10 years	229	21.26%
11 to 20 years	293	27.21%
21 or more years	207	19.22%
**Prior research mentorship training (*n* = 1076)**		
Yes	457	42.47%
No	619	57.53%

a Respondents could select more than one category.

*OPM* = *Optimizing the Practice of Mentoring* (online training module).

#### Engagement and training satisfaction

*OPM* users’ level of engagement with the module differed, but the majority (55%, 577/1049) reported spending 1.5 to 2 hours working through the content (Supplementary Figure 1). A large proportion of respondents (77%, 810/1048) agreed or strongly agreed that “Participating in this course was a valuable use of my time,” with a mean score of 3.92 (*SD* = 0.91) on a 5-point scale. Similarly, 69% (727/1049) of respondents indicated they were likely or very likely to recommend the course to a colleague. The mean score for this item was 3.79 (*SD* = 0.97) on a 5-point scale.

#### Self-appraised mentorship skills, confidence, and overall quality

*OPM* users reported significant pre-to-post gains in all 11 of the core content skill areas assessed (*p* < .001; Table 2). On a 7-point scale, their mean perceived skill levels before module completion (assessed retrospectively) ranged from 3.98 to 4.63, and increased to a range of 5.18 to 5.70 after module completion. Participants also reported significant improvements in their mean ratings for perceived overall quality of mentoring that they can provide, their confidence in mentoring, and the degree to which they feel they are meeting their mentees’ expectations (*p* < .001; Table 2).

**Table 2.** Self-reported gains in mentoring skills, quality, and confidence, and in perceived effectiveness at meeting mentee expectations, after completion of *OPM* training

Item	N	Before training mean (SD)	After training mean (SD)	Mean difference (SD)*	95% CI of the difference	
					Lower	Upper
Skills^a^						
Defining the value of mentoring for research career development	1036	4.63 (1.27)	5.70 (0.93)	1.07 (0.99)	1.01	1.13
Recognizing the pros and cons of different mentoring models	1032	4.16 (1.37)	5.64 (0.93)	1.49 (1.24)	1.41	1.56
Fulfilling the career-enhancing functions of being a research mentor	1032	4.59 (1.32)	5.67 (0.96)	1.08 (1.04)	1.02	1.15
Fulfilling the psychosocial functions of being a research mentor	1026	4.46 (1.34)	5.50 (1.01)	1.04 (1.02)	0.98	1.10
Applying a proactive, structured approach to mentoring	1026	4.23 (1.37)	5.55 (0.98)	1.33 (1.13)	1.26	1.40
Navigating the different phases of a mentoring relationship	1025	4.15 (1.42)	5.48 (1.00)	1.33 (1.17)	1.26	1.41
Applying specific strategies to enhance the quality of my mentoring relationships	1028	4.12 (1.32)	5.40 (0.98)	1.28 (1.08)	1.22	1.35
Addressing challenges that might arise in my research mentoring relationships	1018	4.18 (1.34)	5.40 (1.00)	1.22 (1.09)	1.15	1.29
Engaging in difficult conversations with my mentees	1021	4.07 (1.45)	5.18 (1.15)	1.11 (1.09)	1.04	1.17
Routinely reflecting on and adapting my mentoring practices	1023	4.15 (1.40)	5.35 (1.02)	1.20 (1.11)	1.13	1.27
Leveraging existing resources and tools to support my mentoring practices	1017	3.98 (1.38)	5.30 (1.08)	1.32 (1.20)	1.24	1.39
Overall Mentoring Quality^b^	1027	4.60 (1.15)	5.47 (0.90)	0.87 (0.90)	0.82	0.93
Confidence in Mentoring^b^	1030	4.63 (1.19)	5.50 (0.92)	0.87 (0.88)	0.82	0.92
Meeting Mentees’ Expectations^c^	1027	4.64 (1.09)	5.33 (0.89)	0.68 (0.91)	0.63	0.74

* *p* < 0.001 for the difference in mean scores between pre and post training for all items using paired *t*-tests.

a Retrospective pre-post (1–7 scale: 1 = *not at all skilled*, 4 = *moderately skilled*, 7 = *extremely skilled*).

b Retrospective pre-post (1–7 scale: 1 = *very low*, 4 = *average*, 7 = *very high*).

c Retrospective pre-post (1–7 scale: 1 = *not at all*, 4 = *moderately*, 7 = *completely*).

*OPM* = *Optimizing the Practice of Mentoring* (online training module).

#### Intention to change mentoring behaviors

A high proportion of respondents (83%, 864/1044) reported making or intending to make changes in their mentoring relationships as a result of participating in the training. Of these, 688 individuals offered an open-ended description of their behavioral changes. Our word query analysis of these free-text responses identified several commonly used words that both aligned with the training module’s content and reflected behavioral actions (Table 3). Among the high-frequency words meeting both of these criteria, *expectation* was the most commonly cited (*n* = 153 unique respondents). These responses reflected *OPM* users’ intentions to more clearly communicate, align, and address *expectations* within their mentoring relationships. Other frequently referenced stem words were indicative of respondents’ intentions to use mentoring *plans* (*n* = 84), to pay more attention to *goal* setting (for the mentoring relationship and/or for the mentee’s career; *n* = 77), and to adopt a more *structured* approach to mentoring (*n* = 72). Some of the less commonly cited words, but all of which directly align with *OPM* module content, indicated respondents’ intentions to improve *communication* with their mentees (*n* = 45), to engage in more *reflection* about their approaches to mentoring and the impact of their approaches on mentees (*n* = 43), and to increase their *support* of mentees (*n* = 28).

**Table 3.** Categories of intended behavioral change prompted by participation in *OPM* training

Stem word (Number of unique respondents)	Word query definition	Example quotes
1. Expectations (153)	Respondents noted plans to more clearly articulate or communicate expectations, to better align expectations, to develop or write their expectations, or to revisit expectations.	“I will also be more explicit about my expectations and ask about mentee expectations during the “development” phase of the relationship. This way I hope to avoid entering mentoring relationships in which expectations are not easily aligned.” “Setting expectations for mentees. And more actively seeking to know what their career expectations are.”
2. Plan (84)	Respondents indicated their intentions to create new or use existing written plans such as a mentoring plan, mentoring agreement, or individual development plan.	“I plan to take a more interactive approach and more actively engage my mentees in developing a structured plan to improve success and career development.” “I plan to make more specific mentoring action plans and be more consistent in discussing things adaptively with trainees.”
3. Goals (77)	Respondents described plans to identify/set goals including short and long term career goals, check in on progress towards goals, or help their mentees achieve goals; a few noted plans to share their own mentoring goals with mentees.	“More direct discussion with mentees about long term goals.” “Discuss career goals with mentee earlier in relationship.” “When goals aren”t met, thinking about whether interests have diverged or the goals weren’t clear.”
4. Structure (72)	Respondents referred to adding more structure to their mentoring interactions. […]	“I will try to be more thoughtful and more organized about how to approach mentoring in a structured way, rather than just engaging in it without a clear plan of action.” “I plan to use a more deliberate, structured approach that draws on resources and helping mentees grow in independence.”
5. Meetings (66)	Respondents noted plans to change their approach to meetings with their mentees including alterations to structure, agendas, preparation, and frequency for both individual and research team meetings.	“I want to incorporate meeting agendas and meeting reports.”
6. Communication (45)	Respondents noted plans for more communication, clearer communication, and open and better communication.	“More communication about our relationship.” “I will try to increase communication/trust by active listening […]”
7. Reflection (43)	Respondents described plans for more self-reflection on mentoring, on their mentoring relationship, and on their mentoring practices.	“I will apply more self-reflection, particularly about my bias. I will be more aware of the different stages of mentoring, applying principles to make it better at every stage.” “Do more active listening. Seek out perspectives of mentees. Do more self-reflection.”
8. Proactive (41)	Respondents articulated plans to be more proactive in their mentoring generally and across a range of specific topics.	“More proactive approach to identifying problems and solutions with direct input from the mentee.” “I will be more proactive in engaging mentees about their lives outside of the lab and reflect on how that may impact the mentoring required.”
9. Support (28)	Respondents described intentions to increase and provide more direct support for mentees; several noted plans to create high challenge / high support environments.	“I intend to be more explicit in my support - including explicitly acknowledging mentee’s contributions and efforts.” “The balance between challenge and support is one that I would like to develop more.”

### Module reach: total registrant data

The evaluation results described above were drawn from a subset of total *OPM* registrants. Our examination of the full registrant dataset identified 4,011 unique individuals who registered for *OPM* from its initial launch on October 17, 2012, through June 1, 2022. Growth over time in unique registrants per year and in cumulative number of registrants by year is illustrated in Supplementary Figure 2. Registrants included 663 (16.5%) individuals from the University of Minnesota and 3,348 (83.5%) from other institutions (Supplementary Table 1). More than 650 institutions are represented in this sample. We were able to confirm that 2,646 registrants (66%) were employed at one of 80 past or present CTSA-affiliated institutions. Most registrants were higher education faculty members and/or administrators, with the remainder identifying as a student or fellow, health professional, or other job category. Approximately half (52%) indicated that their engagement with the module was required by their institution or program, 37% were proactively using it for their own professional development, and 13% were reviewing the module for possible future use in their own institutions (Supplementary Table 1).

## Discussion

Within clinical and translational science and related fields, the responsibilities of research mentorship are recognized as a constellation of competencies that can be honed through evidence-based professional development programs [6,21,25–28]. Our usage and evaluation data for the online module, *OPM*, demonstrate that this relatively brief mentor training mechanism – designed for self-paced, interactive, asynchronous learning – has broad appeal, yields positive gains in specific mentorship skills and other learner outcomes, and prompts behavior change intentions aimed at improving the quality of mentoring relationships.

In support of *OPM’s* efficacy as a training modality, individuals in our large evaluation sample reported post-training gains in each of the skill areas covered by the module. The first two *OPM*-specific skills (“Defining the value of mentoring for research career development” and “Recognizing the pros and cons of different mentoring models”) could be considered to be more reflective of knowledge gains. However, the remaining nine reflect categories of behaviors that either directly engage mentees (e.g., “Applying a proactive structured approach to mentoring”) or support users’ professional growth as mentors (e.g., “Routinely reflecting on and adapting my mentoring practices”). In comparison to previously reported pilot work involving *OPM* [18], the current sample was substantially larger and more diverse with respect to career stages and years of mentoring experience, thereby enhancing the generalizability of learner outcomes that we report.

Gains were also observed in the three general measures of perceived mentoring confidence, overall quality of mentoring, and effectiveness at meeting mentees’ expectations. For the latter two metrics, the measured gains for *OPM* users were of similar magnitude to those reported for individuals who participated in at least 6 hours of *Entering Mentoring*-based workshops [23]. These findings offer additional support for *OPM’s* value as a standalone, asynchronous online training option – one that requires a modest amount of time to complete and can be accessed at a time and place convenient to individual learners. In the absence of data from a head-to-head trial, direct comparisons of *OPM* outcomes to those of other interventions should be interpreted cautiously. In terms of training duration alone, *OPM* is more comparable to the “low dose” (<4 hours) variations of *Entering Mentoring* workshops that have been previously found to be effective, though to a lesser degree on some measures than higher dose training iterations [22].

Engagement with *OPM* prompted a clear intention among a large proportion of users (83%) to adapt their mentoring in direct response to what they had learned. This metric compares favorably to that reported by participants in *Entering Mentoring* across different modalities and dosages (91%, unpublished data) and by participants in the advancing inclusive mentoring (AIM) program (90%) developed at California State University Long Beach [29,30]. The intended behavioral changes noted by *OPM* users largely coalesced around the themes of aligning expectations, goal setting, and other planning-focused tasks reflective of a proactive approach to structuring their interactions with mentees. These topics are extensively addressed in the module and reinforced through checklists, tools, and suggested conversation prompts.

*OPM* was developed in partnership with experts in instructional design and e-learning. Their input ensured that users are given frequent opportunities for self-reflection and real-time engagement with the material (via brief exercises, mini-surveys, and simulated discussions). We posit, based on informal feedback from users, that features such as these have contributed to *OPM’s* appeal and impact as an independent mentor training program.

We also know from work by us and others that online, asynchronous approaches to mentor training offer versatility in how they are implemented [18,29]. Although they cannot replicate the interpersonal discussions that might take place within a well-facilitated synchronous workshop setting, they can be leveraged to prepare mentors to more fully engage in meaningful group work. As noted in this article’s introduction, we have done this successfully within the hybrid mentor training approach of the University of Minnesota’s Mentoring Excellence Training Academy [18]. Academy participants exhibited knowledge gains and intention-to-change mentoring practices after completion of *OPM* alone; these gains were enhanced after completion of subsequent in-person workshops. In focus groups, mentors said they valued the synergy of the blended learning format, noting the unique strengths of each modality and the benefits of completing a foundational online module before in-person engagement. Across 6 more recent cohorts of the Academy, 82% of participants somewhat or strongly agreed that completion of *OPM* helped prepare them to engage in the facilitated workshops (unpublished data). Other examples of *OPM’s* successful integration into multimodal mentor training initiatives include the University of Wisconsin’s Building Equitable Access to Mentorship (BEAM) program [31], Washington University’s Mentored Training for Dissemination and Implementation Research in Cancer (MT-DIRC) program [32,33], and the Howard Hughes Medical Institute’s Gilliam Fellows Program [34]. While individual programs can design their own mode of integration, *OPM’s* embedded reflection questions, which can be printed and saved by users, offer one simple option for organizing discussion groups that build on the module’s content and support communities of practice.

The full registration data for *OPM’s* first decade of public availability illustrate its widespread reach (over 4,000 unique registrants, two-thirds of whom are affiliated with CTSA hub institutions) and consistent growth (an average of 379 new users/year). At the University of Minnesota, *OPM* training is required for all faculty who mentor trainees in any CTSI-supported program, but users at this institution reflect less than 20% of registrants. These national usage outcomes are consistent with the module creators’ goal of broad dissemination to support mentor professional development.

Several factors are likely to have influenced the ongoing expansion of *OPM* enrollment. First, as shown by our data, satisfaction ratings for the training were generally favorable. Second, information about the module has been disseminated to target audiences through multiple modalities (e.g., a published article [18], the national academies report on *The Science of Effective Mentorship in STEMM* [6], websites for national organizations such as NRMN and CIMER, and multiple invited national presentations). Third, the COVID-19 pandemic heightened institutions’ need for virtual learning, which likely enhanced the attractiveness of online training options. Fourth, over half of *OPM* registrants indicated that module completion was “required by my institution, department, or program,” thereby driving enrollment.

There are limitations to our analyses of *OPM* evaluation data. Our results reflect those of approximately 60% of total registrants from the 3-year evaluation time period. Because the module was purposefully designed to allow for non-linear progression through the material, we are unable to determine whether the remaining registrants completed the course and chose not to submit the survey, or did not finish the course. Our findings could be positively skewed if survey respondents had more favorable views of the course or differed in other substantial ways than non-respondents. It is possible that some users completed the evaluation survey after only minimal engagement with *OPM.* This would make our positive findings a conservative estimate of the module’s impact. The evaluation survey does not capture longitudinal behavior change; the available data are limited to respondents’ immediate post-training intentions to apply the module’s content to their future mentoring practices. Finally, this report relies on mentor self-report data and does not capture the perspectives of mentees whose mentors participated in the *OPM* module. We know from other research that synchronous, in depth, mentor training approaches can have a positive impact on mentees [21,34–38]. Future randomized trials of asynchronous training models such as *OPM* – with enrollment of mentor-mentor dyads and comparison of relevant outcomes for the mentees of trained versus untrained mentors – would be a valuable contribution to the field.

In conclusion, the evaluation and registration data for *OPM* demonstrate its value as an online, asynchronous training tool that can enhance mentorship skills, confidence, and practices across diverse settings. *OPM’s* design offers flexibility in its implementation as a standalone training module, as a prerequisite for more specific or advanced training, or as one component of a multifaceted mentor development program.

## Supporting information

Weber-Main et al. supplementary material Weber-Main et al. supplementary material

## Supplementary material

The supplementary material for this article can be found at https://doi.org/10.1017/cts.2025.84.
