# Supplementary material for: National dissemination of an online research mentor training intervention: Evidence of an asynchronous model to promote learning outcomes and behavior change
Source: J Clin Transl Sci. 2025 Apr 28;9(1):e113. doi: 10.1017/cts.2025.84 (PMC12171925; doi:10.1017/cts.2025.84)
Supplement: Weber-Main et al. supplementary material [file S2059866125000846sup001.docx]

**SUPPLEMENTARY MATERIAL**

**Supplementary Table 1.** Institutional affiliation, job category, and reason for module registration for all *OPM* registrants (n=4,011) from the module’s launch on October 17, 2012 through June 1, 2022. The subsample of participants whose evaluation surveys were used for our outcomes analyses (n= 1,124) are taken from this parent group.

| **Survey Item** | **Number** | **Percentage** |
| --- | --- | --- |
| **Home institution** |  |  |
| University of Minnesota | 663 | 16.5% |
| Other | 3,348 | 83.5% |
| **Institution Type** |  |  |
| Higher education | 3109 | 77.5% |
| Hospital or medical center | 645 | 16.1% |
| Other | 247 | 6.1% |
| Missing | 10 | 0.25% |
| **Job Category^a^** |  |  |
| Faculty member | 2936 | 73.0% |
| Administrator | 532 | 13.3% |
| Student or fellow | 442 | 11.0% |
| Health professional | 401 | 10.0% |
| Other | 213 | 5.3% |
| Missing | 11 | 0.27% |
| **Reason for registration^a^** |  |  |
| Required by my institution, department, or program | 2102 | 52.4% |
| My own professional development | 1477 | 36.8% |
| Reviewing module for possible use at my institution, department, or program | 527 | 13.1% |
| Other | 106 | 2.6% |
| Missing | 30 | 0.75% |

**^a^Respondent could select more than one option**

**
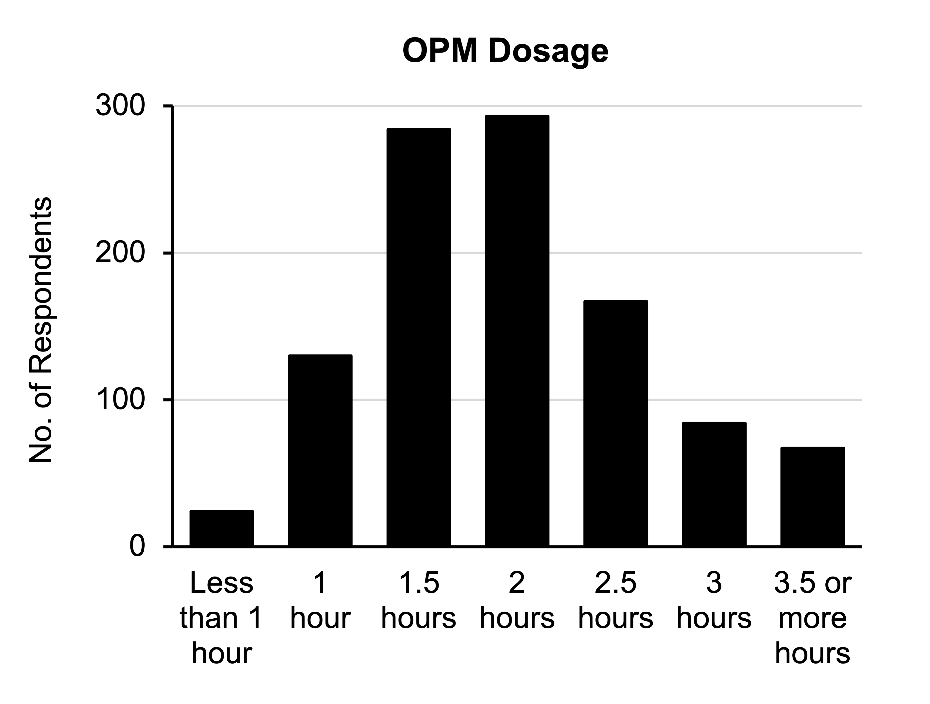
**

**Supplementary Figure 1.** Time spent engaging with the online training module, *Optimizing the Practice of Mentoring (OPM).* Data are presented for 1,049 evaluation survey respondents.

**
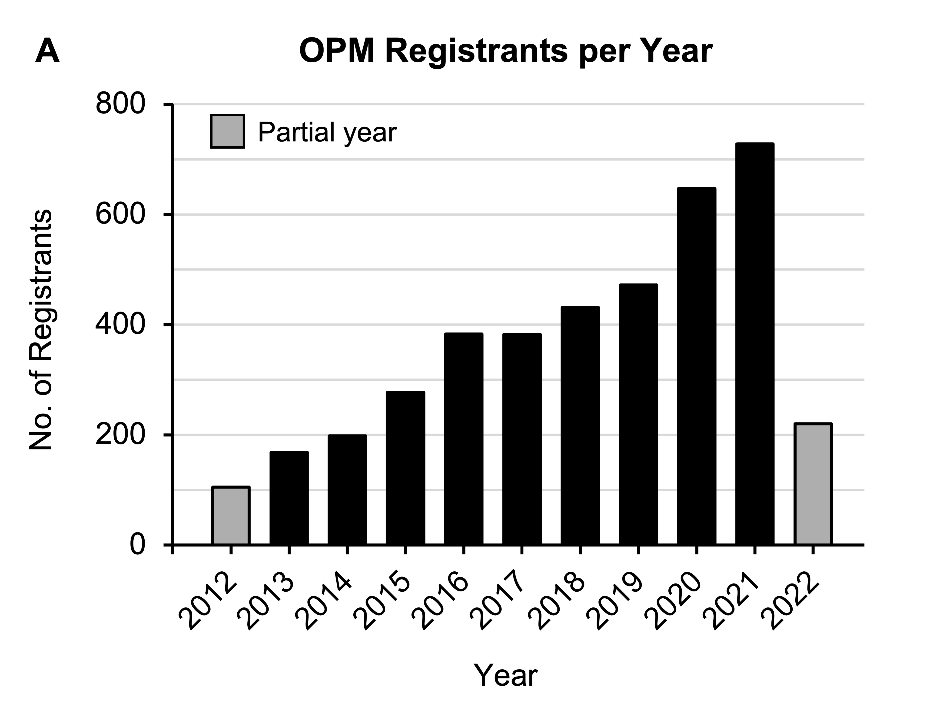
**

**
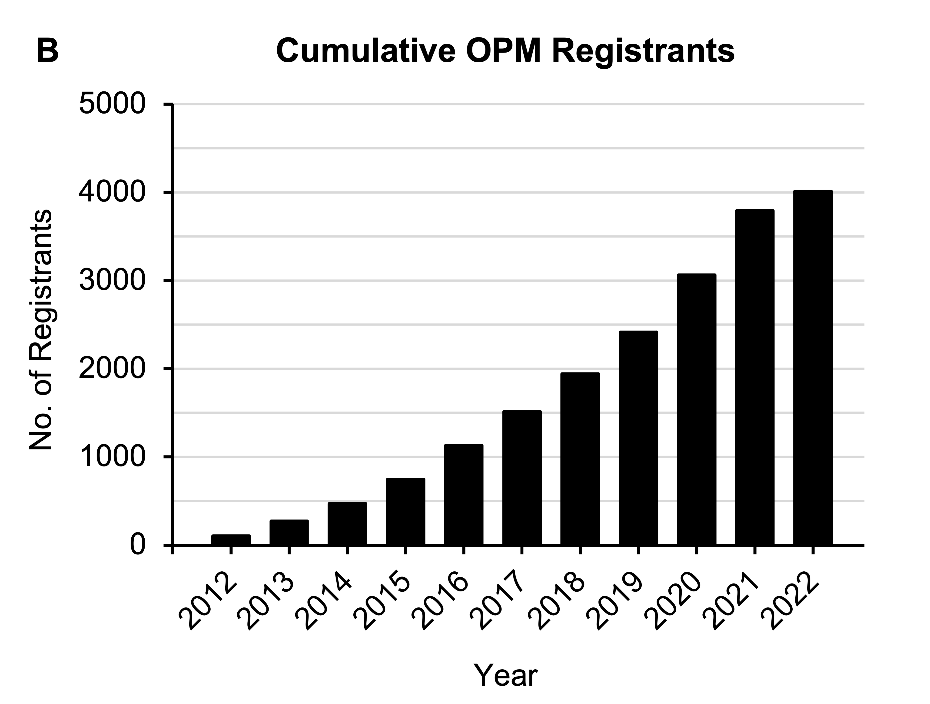
**

**Supplementary Figure 2.** **Growth in usage of the online training module, *Optimizing the Practice of Mentoring (OPM).*** Number of unique registrants per year (panel A) and cumulative registrants over time (panel B) are graphed for the period beginning October 17, 2012 and ending June 1, 2022.
